# Whether the newly modified rhizotomy protocol is applicable to guide single-level approach SDR to treat spastic quadriplegia and diplegia in pediatric patients with cerebral palsy?

**DOI:** 10.1007/s00381-019-04368-w

**Published:** 2019-09-09

**Authors:** Qijia Zhan, Xidan Yu, Wenbin Jiang, Min Shen, Shuyun Jiang, Rong Mei, Junlu Wang, Bo Xiao

**Affiliations:** 1 Department of Neurosurgery, Shanghai Children’s Hospital, Shanghai Jiao Tong University, Shanghai, China; 2 Department of Rehabilitation Medicine, Shanghai Rehabilitation and Vocational Training Center for the Disabled, Shanghai, China; 3 Gait and Motion Analysis Center, Yueyang Hospital of Integrated Traditional and Western Medicine, Shanghai University of Traditional Chinese Medicine, Shanghai, China

**Keywords:** Spastic cerebral palsy, Dorsal rhizotomy, Intra-operative neuroelectrophysiology, Rootlet selection, Outcome

## Abstract

**Purpose:**

Our aim was to test whether the newly modified rhizotomy protocol which could be effectively used to guide single-level approach selective dorsal rhizotomy (SL-SDR) to treat spastic hemiplegic cases by mainly releasing those spastic muscles (target muscles) marked pre-operatively in their lower limbs was still applicable in spastic quadriplegic or diplegic cerebral palsy (CP) cases in pediatric population.

**Methods:**

In the current study, we retrospectively conducted a cohort review of cases younger than 14 years of age diagnosed with spastic quadriplegic or diplegic CP who undergone our modified protocol-guided SL-SDR in the Department of Neurosurgery, Children’s Hospital of Shanghai since July 2016 to November 2017 with at least 12 months post-op intensive rehabilitation program (pre-op GMFCS level-based). Clinical data including demographics, intra-operative EMG responses interpretation, and relevant assessment of included cases were taken from the database. Inclusion and exclusion criteria were set for the selection of patients in the current study. Muscle tone (modified Ashworth scale) and strength of those spastic muscles (muscle strength grading scale), range of motion (ROM) of those joints involved, the level of Gross Motor Function Classification System (GMFCS), and Gross Motor Function Measure 66 items (GMFM-66) score of those cases were our focus.

**Results:**

A total of 86 eligible cases were included in our study (62 boys). Among these patients, 61.6% were quadriplegic. Pre-operatively, almost 2/3 of our cases were with GMFCS levels II and III. Mean age at the time of surgery in these cases was 6.2 (3.5–12) years. Pre-op assessment marked 582 target muscles in these patients. Numbers of nerve rootlets tested during SDR procedure were between 52 and 84 across our cases, with a mean of 66.5 ± 6.7/case. Among those tested (5721 in 86 cases), 47.9% (2740) were identified as lower limb-related sensory rootlets. Our protocol successfully differentiated sensory rootlets which were considered to be associated with spasticity of target muscles across all our 86 cases (ranged from 3 to 21). Based on our protocol, 871 dorsal nerve rootlets were sectioned 50%, and 78 were cut 75%. Muscle tone of those target muscles reduced significantly right after SL-SDR procedure (3 weeks post- vs. pre-op, 1.7 ± 0.5 vs. 2.6 ± 0.7). After an intensive rehabilitation program for 19.9 ± 6.0 months, muscle tone continued to decrease to 1.4 ± 0.5. With the reduction of muscle tone, strength of those target muscles in our cases improved dramatically with statistical significance achieved (3.9 ± 1.0 at the time of last follow-up vs. 3.3 ± 0.8 pre-op), and as well as ROM. Increase in GMFCS level and GMFM-66 score was observed at the time of last follow-up with a mean of 0.4 ± 0.6 and 6.1 ± 3.2, respectively, when compared with that at pre-op. In 81 cases with their pre-op GMFCS levels II to V, 27 (33.3%) presented improvement with regard to GMFCS level upgrade, among which 4 (4.9%) even upgraded over 2 levels. Better results with regard to upgrading in level of GMFCS were observed in cases with pre-op levels II and III when compared with those with levels IV and V (24/57 vs. 3/24). Upgrading percentage in cases younger than 6 years at surgery was significantly greater than in those older (23/56 vs. 4/25). Cases with their pre-op GMFM-66 score ≥ 50 had greater score increase of GMFM-66 when compared with those less (7.1 ± 3.4 vs. 5.1 ± 2.8). In the meanwhile, better score improvement was revealed in cases when SDR performed at younger age (6.9 ± 3.3 in case ≤ 6 years vs. 4.7 ± 2.7 in case > 6 years). No permanent surgery-related complications were recorded in the current study.

**Conclusion:**

SL-SDR when guided by our newly modified rhizotomy protocol was still feasible to treat pediatric CP cases with spastic quadriplegia and diplegia. Cases in this condition could benefit from such a procedure when followed by our intensive rehabilitation program with regard to their motor function.

## Introduction

To reduce muscle tone mainly in particular spastic muscles of lower limbs in spastic cerebral palsy (CP) children via single-level approach selective dorsal rhizotomy (SL-SDR) using a universally applicable rhizotomy protocol still remains challenging [1]. In the current study, we investigated whether our modified rhizotomy protocol-guided SL-SDR which could effectively decrease muscle tone of those particular spastic muscles in hemiplegic cases could be applied as well in spastic diplegic and quadriplegic pediatric CP cases.

## Methods and materials

In the current study, we retrospectively conducted a cohort review in cases younger than 14 years of age diagnosed with spastic diplegic or quadriplegic CP who undergone our modified protocol-guided SDR in the Department of Neurosurgery, Children’s Hospital of Shanghai since July 2016 to November 2017 with at least 12-month intensive rehabilitation program. Clinical data including demographics, intra-operative electromyography (EMG) recordings, and relevant evaluation results of the included cases were taken from the Database of Pediatric Cerebral Palsy Patients in the Rehabilitation Department of both Children’s Hospital of Shanghai and Shanghai Rehabilitation and Vocational Training Center for the Disabled. Diagnosis of spastic CP for these cases was made by a specialized team including specialists in the rehabilitation department of both institutes.

Inclusion criteria for the cases included in the current study were as follows: (1) with diagnosis of spastic diplegic or quadriplegic CP; (2) age at SDR guided by our modified protocol between 3 and 14 years; (3) after SDR, at least 12-month rehabilitation therapy conducted at the rehabilitation department of Children’s Hospital of Shanghai or Shanghai Rehabilitation and Vocational Training Center for the Disabled; and (4) pre-op and follow-up assessment was conducted by the same rehabilitation team. The exclusion criteria included were as follows: (1) combined with other neurological disorders, such as uncontrolled seizures; (2) spasticity due to head or spinal cord trauma; (3) with tendon contractures or joints deformity in the lower extremities; (4) previously operated on with any orthopedic procedures to release spasticity; (5) with moderate to severe mental retardation; and (6) lost to follow-up.

### Evaluation

As a routine, assessment measures for CP children having their rehabilitation therapy conducted in both institutes were as follows: (1) muscle tone (using the modified Ashworth scale) and strength (muscle strength grading scale) of those spastic muscle groups in lower extremities; (2) range of motion (ROM) of those joints involved in lower limbs; and (3) the level of Gross Motor Function Classification System (GMFCS) and Gross Motor Function Measure 66 items (GMFM-66) score. Any muscle of adductors, hamstrings, gastrocnemius, and soleus with its muscle tone ≥ grade 2 revealed in pre-op evaluation would be marked as target muscle. All cases needed to have their head and lumbar MRI without contrast done before SDR.

### Surgical procedure, intro-operative stimulation, and EMG interpretation

Briefly, surgery was performed under general anesthesia with minimum alveolar concentration (MAC) of sevoflurane inhalation at 0.5 and maintenance of body temperature between 36.0 and 37.0 °C. The skin incision was typically at the level of L2. After the single level laminectomy (L2) done, dura incision of 10–12 mm was made. A 5-mm Silastic sheet was placed under all nerve rootlets, including ventral and dorsal ones following the opening of arachnoid membrane in the surgical field. After all nerve rootlets tested and the ones matched our rhizotomy criteria sectioned, the dura and skin incision was closed in layers.

**Fig. 1:**
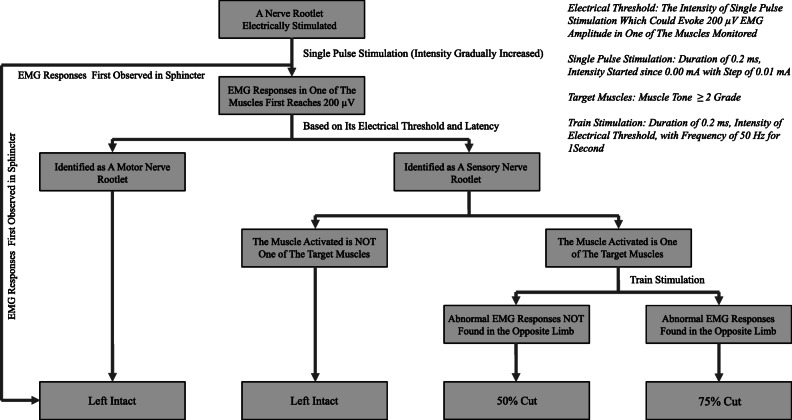
Scheme of our modified rhizotomy protocol

**Fig. 2:**
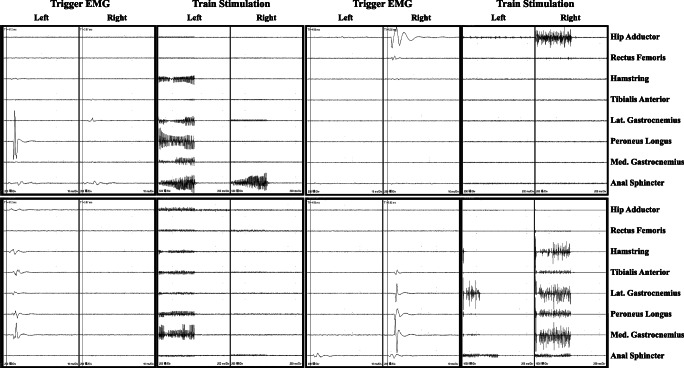
Example of EMG response interpretation. a, Single pulse stimulation elicited EMG responses in Lt. peroneus longus which was not the “target muscle”, the rootlet was left spared; b, EMG responses elicited in Rt. hip adductor which was the “target muscle”, but abnormal EMG responses were not seen on the Lt. side when train stimulation was given, the rootlet was cut 50%; c, EMG responses mainly seen in Lt. gastrocnemius medialis which was the “target muscle” when single pulse stimulation was given, but train stimulation did not elicit abnormal EMG responses on the Rt. side, the rootlet was cut 50%; d, Triggered EMG responses observed in Rt. gastrocnemius medialis which was the “target muscle”, train stimulation did evoke abnormal EMG responses on the Lt. side (mainly the lateral gastrocnemius), the rootlet was cut 75%

EMG recording was conducted in 15 channels, including bilaterally in the hip adductor, hamstring, rectus femoris, medial gastrocnemius, lateral gastrocnemius, peroneus longus, tibialis anterior, and anal sphincter. Stimulation output and EMG recording were achieved using the Cadwell-Cascade Elite monitoring system. Single pulse of electrical stimulation with a duration of 0.2 ms was given to the rootlet tested during surgery with intensity started since 0.00 mA with a step of 0.01 mA. The intensity which could evoke EMG responses with amplitude reaching 200 μV in a particular muscle was defined as “electrical threshold” of that rootlet. Our modified rhizotomy protocol was detailed in Fig. 1. Example of EMG interpretation was shown in Fig. 2.

### Postoperative care and rehabilitation

**Fig. 3:**
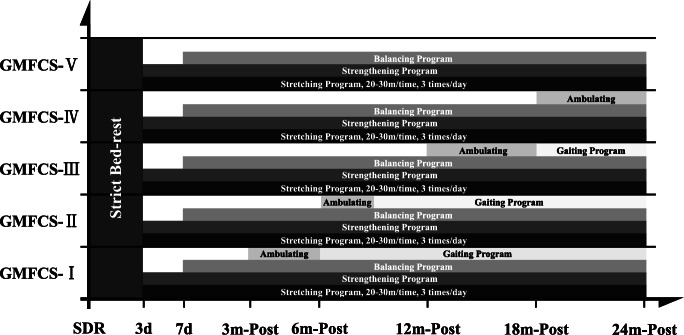
Framework of pre-op GMFCS level–based post-op rehabilitation program

Patients stayed in PICU for one night after the procedure. Bedside rehabilitation program started 3–5 days post-op, mainly including lower extremity’s stretching and strengthening. Usually patients were transferred to outpatient rehabilitation center 3 weeks post-SDR to receive an intensive rehabilitation program. Detailed post-op rehabilitation protocol is depicted in Fig. 3.

### Statistical analysis

Data were analyzed using commercial statistical software (SPSS version 19.0, IBM). To compare intergroup data, appropriate univariate analyses were used: Student’s *t* test for continuous data, the Fisher exact test for binomial categorical data, and the chi-square test for unranked categorical data.

## Results

In a total of 135 cases who undergone our modified protocol-guided SL-SDR, 86 matched our study criteria (62 boys and 24 girls), among which 61.6% (53 cases) were quadriplegic. Pre-operatively, almost 2/3 of our cases were with their GMFCS levels II and III. Mean age at the time of surgery in these cases was 6.2 (3.5–12.0) years. A total of 582 muscles and 413 joints in the lower limbs were marked as target muscles and joints involved in pre-op assessment, respectively. The number of nerve rootlets tested during SDR procedure was between 52 and 84 over our 86 cases with a mean of 66.5 ± 6.7/case. Among those tested (5721 in 86 cases), 47.9% (2740) were identified as sensory rootlets which were related to lower limbs. Based on our protocol, 871 dorsal nerve rootlets determined to be associated with spasticity of those target muscles were sectioned 50%, and 78 were cut 75%. The number of rootlets with their EMG responses matched our rhizotomy criteria varied from 3 to 21 across our cases and was 6.4 ± 4.0, 9.8 ± 4.4, and 12.8 ± 3.2 in cases with their number of target muscle of 3–4, 5–6, and 7–8, respectively. After post-op rehabilitation therapy over a mean duration of 19.9 ± 6.0 months, GMFCS level improved from 3.0 ± 1.0 to 2.6 ± 1.2 (*p* < 0.05), and GMFM-66 score increased at a mean of 6.1 ± 3.2 in our cases (*p* < 0.01). No permanent surgery-related complications were recorded in the current study (Tables 1 and 2).

**Table 1:** General clinical data of 86 cases included in the study

Characteristics	No. (%)^a^
Gender	
Boy	62 (72.1)
Girl	24 (27.9)
Age at surgery, year (mean, SD)	3.5–12.0 (6.2 ± 2.0)
Etiology of spasticity	
Premature	21 (24.4)
Asphyxia	48 (55.8)
Unknown	17 (19.8)
Spastic type	
Quadriplegia	53 (61.6)
Diplegia	33 (38.4)
Target muscles identified at pre-op, number (mean, SD)/case	3–8 (6.8 ± 1.3)
Main Joints involved, number (mean, SD)/case	2–6 (4.8 ± 1.3)
GMFCS level	
At pre-op (mean, SD)	3.0 ± 1.0
Level I	5 (5.8)
Level II	22 (25.6)
Level III	35 (40.7)
Level IV	19 (22.1)
Level V	5 (5.8)
At the last follow-up (mean, SD)	2.6 ± 1.2^#^
Level I	17 (19.8)
Level II	26 (30.2)
Level III	22 (25.6)
Level IV	16 (18.6)
Level V	5 (5.8)
GMFM-66	
At pre-op (mean, SD)	53.0 ± 13.1
At the last follow-up (mean, SD)	59.1 ± 14.9*
Surgery-related complications	
CSF leak or infection	0 (0.0)
Hypoesthesia	0 (0.0)
Hypersensitivity (≤ 2 weeks)	17 (19.8)
Urinary/bowel incontinence	0 (0.0)
Follow-up, month (mean, SD)	19.9 ± 6.0
Additional orthopedic procedures required during follow-up	
Tendon lengthening	2 (2.3)

*SD* standard deviation, *GMFCS* gross motor function classification system, *GMFM* gross motor function measure, *CSF* cerebral spinal fluid

^a^Unless otherwise indicated

^#^Statistically significant difference obtained when compared with pre-op (*p* < 0.05)

*Statistically significant difference obtained when compared with pre-op (*p* < 0.01)

**Table 2:** EMG interpretation and rhizotomy data in the study

Characteristics	Value (mean, SD)
Rootlets stimulated (number)/case	52–84 (66.5 ± 6.7)
Sphincter associated rootlets (number)/case	17–31 (20.1 ± 3.2)
Motor rootlets associated with lower limbs (number)/case	12–18 (14.6 ± 1.3)
EMG threshold (mA)	0.01–0.11 (0.06 ± 0.03)
EMG latency to the Stimulus (ms)	3.00–9.10 (6.01 ± 1.85)
Sensory rootlet associated with lower limbs (number)/case	19–47 (31.9 ± 5.7)
EMG threshold (mA)	0.15–2.00 (0.54 ± 0.31)
EMG latency to the Stimulus (ms)	6.1–17.1 (12.55 ± 2.71)
Rootlets matched our rhizotomy criteria (number)/case	3–21 (11.0 ± 4.2)
Rootlet cut 50%	3–20 (10.1 ± 3.6)
Rootlet cut 75%	0–3 (0.9 ± 1.0)

*EMG* electromyography, *SD* standard deviation, *mA* milliampere, *ms* millisecond

Muscle tone of those target muscles decreased significantly right after SDR and had a tendency to continue to reduce during the follow-up (2.6 ± 0.7, 1.7 ± 0.5, and 1.4 ± 0.5 in all 582 target muscles, pre-op, 3 weeks post-op, and at the time of the last follow-up, respectively). Major improvement of the strength of those target muscles was revealed after intensive rehabilitation therapy when compared with pre-op status with statistical significance achieved (3.9 ± 1.0 vs. 3.3 ± 0.8). ROM of those involved joints improved as well (Table 3).

**Table 3:** Changes of muscle tone, muscle strength of the target muscles, and ROM of the joints involved (passive) pre- and post-op

Measures	At pre-op	3 weeks post-op	At the last follow-up
	Value (mean, SD)	Value (mean, SD)	Value (mean, SD)
Muscle tone (grade)			
Gastrocnemius	3.0 ± 0.7	2.0 ± 0.5*	1.6 ± 0.4*
Soleus	2.6 ± 0.7	1.4 ± 0.4*	0.9 ± 0.6*
Hamstring	2.3 ± 0.5	1.7 ± 0.4*	1.5 ± 0.4*
Hip adductor	2.5 ± 0.5	1.4 ± 0.3*	1.4 ± 0.3*
Muscle Strength (grade)^a^			
Gastrocnemius	3.5 ± 0.7	3.4 ± 0.7	4.2 ± 0.9*
Soleus	3.4 ± 0.8	3.4 ± 0.8	4.0 ± 1.0*
Hamstring	3.1 ± 0.9	3.1 ± 0.9	3.5 ± 1.1*
Hip adductor	3.2 ± 0.8	3.2 ± 0.8	3.5 ± 1.0*
ROM of joints involved (degree)			
Ankle^b^	1.0 ± 3.9	12.6 ± 3.0*	15.9 ± 4.3*
Knee	128.5 ± 6.9	133.6 ± 5.4*	136.1 ± 4.3*
Hip^c^	30.2 ± 3.3	41.0 ± 2.6*	41.4 ± 2.9*

*ROM* range of motion, *SD* standard deviation

^a^Not applicable in 5 cases with their pre-op GMFCS level V

^b^Dorsal flexion

^c^Abduction with hip flexed

*Statistically significant difference obtained when compared with pre-op (*p* < 0.01)

**Fig. 4:**
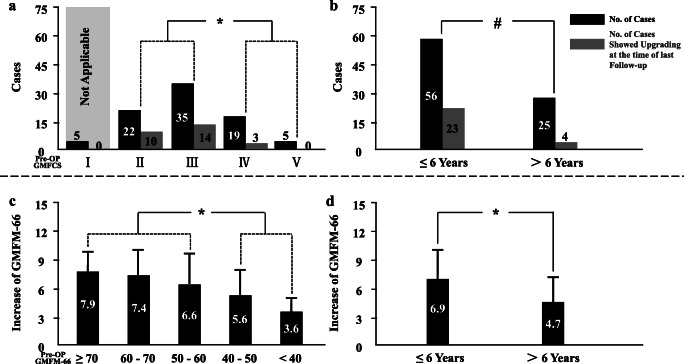
Association between outcomes and pre-op status in our cases. **a** Better results were observed in cases with pro-op GMFCS levels II and III; **b** Cases younger than 6 years had more chance to upgrade (81 cases with their pre-op GMFCS levels II–V); **c** Score increased more in cases with pre-op GMFM-66 ≥ 50 than those with score below; **d** Cases younger than 6 years improved more with regard to score increase of GMFM-66 after SDR. Number sign indicates statistically significant difference obtained with *p* < 0.05. Asterisk indicates statistically significant difference obtained with *p* < 0.01

In 81 cases with their pre-op GMFCS levels II–V, 27 (33.3%) showed improvement with regard to GMFCS level upgrade, among which 4 cases (4.9%) even upgraded over 2 levels. Better results with regard to upgrade in level of GMFCS were observed in cases with pre-op levels II and III when compared with those with level IV and V (24/57 vs. 3/24) with *p* < 0.01. Upgrading percentage in cases younger than 6 years of age at surgery was revealed greater than in older ones (23/56 vs. 4/25 with *p* < 0.05). Cases with their pre-op GMFM-66 score more than 50 had a greater score increase when compared with those less (7.1 ± 3.4 vs. 5.1 ± 2.8 with *p* < 0.01). In the meanwhile, better results were obtained in cases when SDR performed at their younger age, which reached statistical significance with *p* < 0.01 (6.9 ± 3.3 in case ≤ 6 years vs. 4.7 ± 2.7 in case > 6 years) (Fig. 4).

## Discussion

**Fig. 5:**
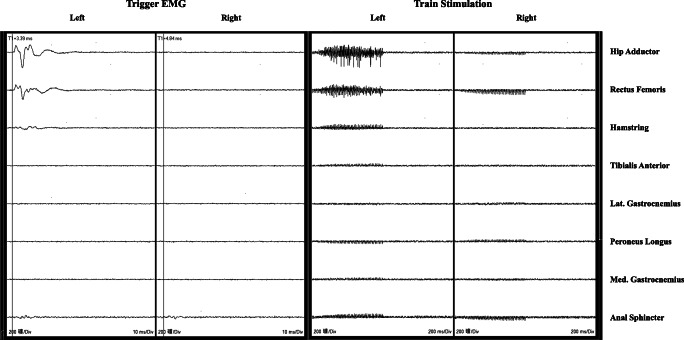
EMG responses obtained in one of our cases with pre-op GMFCS level IV, muscle tone of grade 2 in his Lt. rectus femoris. EMG responses were observed mainly in Lt. hip adductor (one of the target muscles in this case), which met our rhizotomy criteria. The rootlet was cut based on our protocol. In the meanwhile, the EMG responses were seen in Lt. rectus femoris as well. Its muscle tone decreased to grade 1.5 right after the surgery

Preliminary results of our modified rhizotomy protocol-guided SL-SDR to decrease muscle tone of those what we called “target muscles” which were marked during pre-op assessment in spastic hemiplegic CP cases were very encouraging [2]. With the reduction of muscle tone of spastic muscles on their affected lower limbs, their motor function improved dramatically after post-op rehabilitation therapy. Spasticity of lower extremities in spastic diplegic and quadriplegic cases usually affects muscles of adductor, hamstring, gastrocnemius, and soleus with bilateral involvement in a varied degree [3–6]. Besides the spasticity found in these major muscles, muscle tone prominent increase in rectus femoris is observed as well in some of spastic CP children. During the stage of protocol modification, we had a long discussion with rehabilitation specialists both in our hospital and in the rehabilitation center which were in collaboration with us. The major reason that eventually convinced us to exclude rectus femoris from our target muscle list was the concern of its tension for weight bearing [7, 8]. We were afraid of the deterioration in GMFCS level occurred in those cases after our procedure. In the current study, muscle tone ≥ grade 2 in rectus femoris was observed in 4 cases in their pre-op assessment. All these cases were with their pre-op GMFCS levels III to V. Interestingly, reduction of its muscle tone at an average of 0.5 grade was observed 3 weeks post-op in these cases, though SDR procedures in these cases strictly followed our rhizotomy protocol. We considered that such a muscle tone decreases in rectus femoris might be attributable to two factors: (1) cases with prominent spasticity on their rectus femoris were quite severe. Spasticity in those cases was usually global. Tone decrease in those target muscle groups after SL-SDR would somehow positively influence the muscle tone in rectus femoris; (2) hip adductor was identified as our “target muscle” in all of these cases. EMG responses that first appeared in hip adductor and having reached our rhizotomy criteria actually could also be observed in rectus femoris. Therefore, cutting of such rootlet might also result into the reduction of its muscle tone after the procedure (Fig. 5). The slight improvement of muscle tone in rectus femoris in these cases was quite beneficial for the following post-op standing program for these cases. Nevertheless, the justification of its exclusion from the target muscle list requires to be tested and validated in the following studies with more cases and longer follow-up.

Like how it was applied in hemiplegic cases, our modified rhizotomy protocol was quite applicable as well when it was used to guide SL-SDR to treat CP cases with spastic diplegia and quadriplegia with relatively simple intra-operative EMG interpretation. Among 5721 nerve rootlets tested in all cases in the current study, our protocol successfully identified 949 rootlets considered to be associated with spasticity of 582 target muscles marked pre-operatively, which ranged from 3 to 21 over 86 cases. The number of rootlets that matched our modified rhizotomy criteria increased in cases when their pre-op marked target muscle number increased (6.4 ± 4.0, 9.8 ± 4.4, and 12.8 ± 3.2 in target muscle number of 3–4, 5–6, and 7–8, respectively). Such results suggested that our modified rhizotomy protocol was feasible when it guided SL-SDR to release spasticity of the lower limbs in diplegic or quadriplegic cases.

Muscle tone reduction of those target muscles (1.0 ± 0.6 grade in 582 muscles) was satisfactory 3 weeks after SL-SDR, without impairment of their muscle strength observed generally in our cases. In our study, muscle tone decrease had a tendency to continue (0.3 ± 0.4 grade) over the following 19.9 months, which was consistent with previous studies when SDR was guided by the traditional EMG response grading system [6, 9–12]. Significant improvement of spasticity in lower the limbs after our SL-SDR procedure created a better condition for the following intensive rehabilitation program. After a mean duration of 19.9 months of rehabilitation therapy, along with the major strength increase of those 582 target muscles (0.5 ± 0.5 grade), GMFCS level and GMFM-66 score of our cases improved dramatically (0.4 ± 0.6 and 6.1 ± 3.2, respectively). In CP population, GMFM-66 score hardly improves more than 3.0/year when a child is older than 6 years of age treated using the conservative measures [13]. Therefore, such a score increase obtained in our study when SL-SDR was guided using our modified rhizotomy protocol could be taken as a favorable outcome. Similar results were reported from other centers when SDR was performed in the traditional manner [14–17]. Further investigation in our study revealed that cases when SDR performed to younger than 6 years old had much better outcomes than when performed to older ones, no matter to assess them using GMFCS level or GMFM-66 score. These results were comparable to those SDR managed via the traditional way. Not surprisingly, like what earlier studies concluded [1, 14, 15, 17–19], our data showed as well that mild cases improved much better than those severe ones. Cases with their pre-op GMFCS level of II and III had more chance to have their level upgrade than those with IV and V (24/57 vs. 3/24) after the rehabilitation therapy. Interestingly, when we used GMFM-66 score to assess the outcomes of our cases, we found that, after post-op rehabilitation therapy, score increased more in cases having greater pre-op GMFM-66 scores (Fig. 4c). Statistical significance was obtained with *p* < 0.01 when our cases were categorized into two groups (pre-op GMFM-66 scores more than 50 vs. less). When we used score of 60 to divide them, significant difference between two groups still achieved with *p* < 0.05. We considered that two factors might contribute to such a phenomenon: (1) relatively restricted brain damage in mild cases, leaving more motor control involved neuronal network intact; (2) mild cases might be more cooperative during the post-op rehabilitation program, thus having better outcomes. Traditionally, the purpose of treatment for CP cases with their GMFCS levels IV and V was to ease the burden of caretakers of those children and to reduced pain caused by severe spasticity. In the current study, we surprisingly found that 3 cases with their pre-op level IV presented improvement of GMFCS level after a duration of almost 2 years of post-op rehabilitation program. Whether such results were associated with their age (SL-SDR performed around the age of 5 years) and relatively high pre-op GMFM-66 score (above 45 in all) requires to be further investigated in the future.

In the current study, we found that EMG responses of most of those sectioned dorsal rootlets reached the traditional rhizotomy criteria (EMG response grading 3+ or 4+), which means that when SL-SDR is performed in the traditional manner, those rootlets would be cut as well. However, there were up to 35% of rootlets in which EMG responses matched our rhizotomy criteria that did not reach 3+ or 4+ of the traditional grading system. About 7% of rootlets tested during surgery reached the rhizotomy criteria of the traditional one that failed to meet ours. Such phenomenon suggested that dorsal rootlet selection during SDR surgery was quite different when EMG responses were interpreted in a different way. Intro-operative EMG responses are considered to reflect neuroelectrophysiological characteristics of neuronal circuits in the spinal cord of spastic CP children. Though our results suggested that SL-SDR could mainly reduce muscle tone of those particular spastic muscles in the lower limbs of CP children when EMG interpreted in our way, the exact relations of EMG responses evoked via dorsal rootlet stimulation with clinical status of spasticity in CP cases still remained unclear. Therefore, further investigation in the following studies to address how neuroelectrophysiological circuits in the spinal cord exactly work in CP children are required.

In the current study, we found that 2 cases that experienced gastrocnemius weakness on one side 3 weeks after the surgery. Muscle strength decreased over one grade when its muscle tone decreased from grade 4 to 2 in both cases. At the time of the last follow-up, improvement was observed with muscle strength recovering to its pre-op status after rehabilitation therapy in these two cases. Muscle weakness after SDR was frequently reported in the literature [20, 21]. The exact mechanism beneath such phenomenon still remains unclear. Whether it is due to the input significant decrease from muscle spindles resulting into initial motor control difficulty requires to be investigated in the future.

Several shortcomings existed in the current study. In addition to its nature of retrospective cohort review performed in one center, the heterogeneous nature of CP may affect the reliability of our conclusions, though patient selection criteria were set. Second, the follow-up of this study is still short. Whether spasticity would recur in more cases in the future remains unclear. However, since this is the first study trying to propose a standardized rhizotomy protocol which could be universally applied in SL-SDR to treat all types of spastic CP cases via decreasing muscle tone mainly in particular muscles, we hope our study could provide an approach when treating spastic CP patients. In the meanwhile, EMG recordings interpreted in SL-SDR have the potential to help clinicians to understand further about the neuroelectrophysiological characteristics of the spinal cord in CP cases.

## Conclusion

Our modified rhizotomy protocol was applicable to guide SL-SDR to treat pediatric cases with spastic diplegia and quadriplegia. Its efficacy to decrease muscle tone of particular muscles in our cases was favorable. With the significant reduction of muscle tone right after SL-SDR, motor function improved dramatically after a mean duration of 19.9 months of rehabilitation program in our cases.
